# Self-Healing Supramolecular Hydrogels with Antibacterial Abilities for Wound Healing

**DOI:** 10.1155/2023/7109766

**Published:** 2023-02-09

**Authors:** Zhiwu Hong, Lei Wu, Zherui Zhang, Jinpeng Zhang, Huajian Ren, Gefei Wang, Xiuwen Wu, Guosheng Gu, Jianan Ren

**Affiliations:** ^1^Research Institute of General Surgery, Jinling Hospital, Nanjing Medical University, Nanjing, China; ^2^Department of General Surgery, Anhui No. 2 Provincial Peoples' Hospital, Hefei, Anhui, China

## Abstract

Wound healing due to skin defects is a growing clinical concern. Especially when infection occurs, it not only leads to impair healing of the wound but even leads to the occurrence of death. In this study, a self-healing supramolecular hydrogel with antibacterial abilities was developed for wound healing. The supramolecular hydrogels inherited excellent self-healing and mechanical properties are produced by the polymerization of N-acryloyl glycinamide monomers which carries a lot of amides. In addition, excellent antibacterial properties are obtained by integrating silver nanoparticles (Ag NPs) into the hydrogels. The resultant hydrogel has a demonstrated ability in superior mechanical properties, including stretchability and self-healing. Also, the good biocompatibility and antibacterial ability have been proven in hydrogels. Besides, the prepared hydrogels were employed as wound dressings to treat skin wounds of animals. It was found that the hydrogels could significantly promote wound repair, including relieving inflammation, promoting collagen deposition, and enhancing angiogenesis. Therefore, such self-healing supramolecular hydrogels with composite functional nanomaterials are expected to be used as new wound dressings in the field of healthcare.

## 1. Introduction

The skin is the largest protective organ in the body. When the integrity of the skin is compromised, many problems follow, especially infections, which are common in clinical medicine [1–8]. The current common means of dealing with infections is the use of antibiotics, which still faces an increase in bacterial resistance and even leads to treatment failure [9–16]. In recent decades, hydrogels that promote tissue regeneration or allow for tissue replacement have driven the development of some surgical treatments, such as wound dressings, sutures, and hemostatic agents [17–25]. Many materials have been used to develop hydrogels, including natural sources of chitosan, hyaluronic acid, sodium alginate, and synthetic acrylamide, polyvinyl alcohol, and poly (lactic-co-glycolic acid) [26–30]. Hydrogels have promising applications in drug delivery, dressings, and tissue adhesives due to the advantages of native tissue mimicry and biocompatibility [31–36]. Although hydrogels offer many useful properties, these existing hydrogels are limited in their application by problems such as inhomogeneous network structure and permanent covalent cross-linking, resulting in poor mechanical properties and inability to adapt to the dynamics of tissue regeneration and integration with the host. Consuequently, novel materials with excellent mechanical strength produced by dynamic covalent crosslinking are highly desirable for infected wound dressings.

In this study, we achieved this goal by employing Ag NPs as antimicrobial adjuvants to be integrated into the self-healing supramolecular hydrogel to enrich its function as a wound dressing. We presented a photoinitiated self-healing hydrogel with good stretchability and tensile strength using N-acryloyl glycinamide. Self-healing supramolecular hydrogels have been widely used to develop highly functional biomaterials due to their advantages in reversibly cross-linked polymer network of the hydrogel, possessing great potential for realizing yet to be clinically translated tissue engineering therapies [37–41]. Despite the unique advantages of self-healing supramolecular hydrogels in terms of desirable mechanical properties and long-term maintenance of their function, the lack of targeted functionality for biomedical applications still needs to be addressed. The Ag NPs have been widely used in medical, food, electronic, and other fields because of their high safety, good durability, and no drug resistance [42–46]. With the function of the N-acryloyl glycinamide hydrogel (NH), it was found that the prepared hydrogel demonstrated high tensile strength, large stretchability, and good self-healing. Besides, with the function of Ag NPs, the hydrogel possessed good antimicrobial properties. And the biocompatibility of the hydrogels had also been demonstrated. Furthermore, the in vivo experiments showed the hydrogels could significantly promote wound repair, including relieving inflammation, promoting collagen deposition, and enhancing angiogenesis. These features indicate the hydrogels hold promising potential as wound dressings for tissue regeneration.

## 2. Materials and Methods

### 2.1. Materials

Silver nanoparticles (99.5%, 60–120 nm), N-acryloyl glycinamide monomer (98%), and photoinitiator Irgacure 2959® were bought from Sigma-Aldrich (St. Louis, MO, USA) and used without further purification. All other reagents were analytically pure or higher and used as received. The 8–12-week-old male Sprague-Dawley rats were provided by the animal management department of Jinling Hospital, Nanjing University. All animal experiments were performed under the approval of the Animal Investigation Ethics Committee of the Jinling Hospital, and the guidelines of the Guide for the Care and Use of Laboratory Animals were strictly followed.

### 2.2. Methods

#### 2.2.1. Preparation and Characterization of the NH Hydrogels

N-acryloyl glycinamide monomer was first dissolved in deionized water to form a homogeneous and clear solution. Then, photoinitiator (0.5 wt%) and Ag NPs of different concentrations were added into the solution. The mixed solution was then treated in a water bath at 50°C for 10 min to fully dissolve the photoinitiator. Subsequently, the mixed solution was subjected to ultrasonic treatment for 5 min to fully disperse the silver nanoparticles. Finally, the mixed solution is subjected to ultraviolet (UV) irradiation for several minutes to form the NH hydrogel. And the NH hydrogels were labeled according to the different content of Ag NPs in the formation of hydrogel. For example, NH (0) indicates that the Ag NPs content in the hydrogel is 0 *μ*g/ml, NH (200) indicates that the Ag NPs content in the hydrogel is 200 *μ*g/ml, and so on.

In addition, the obtained NH hydrogels were characterized by field emission scanning electron microscopy (SEM) to observe the microstructure of hydrogels. And, a series of mechanical tests of the NH hydrogels, including tensile and compression tests, were performed by MultiTest-i.

#### 2.2.2. Self-Healing Performance of the NH Hydrogels

The NH hydrogels were prepared in a specially designed rectangular mold. Then, the prepared NH hydrogels were divided into two parts of close size, respectively. The two parts of the different hydrogels were reassembled into a new hydrogel block. The obtained hydrogel blocks were fixed in a mold, encapsulated, and processed at elevated temperatures for several minutes. After this, the hydrogel blocks were removed and placed at room temperature to cool. Finally, the reassembled hydrogel blocks were mechanically characterized by MultiTest-i.

#### 2.2.3. Antibacterial Activity of the NH Hydrogels

The antibacterial activity of NH hydrogels was evaluated by agar flat dish diffusion method and live-dead staining. As reported in previous studies [47–49], Gram-negative Escherichia coli and Gram-positive Staphylococcus aureus were suggested and used as model strains. Firstly, the recovered bacteria were scraped into pure phosphate-buffered saline (PBS) solution and shaken to obtain a homogeneous bacterial suspension. Subsequently, the different bacterial suspensions were evenly applied to new agar dishes by sterile cotton swabs, respectively. At the same time, different NH hydrogels were placed in the centre of the agar dishes coated with bacterial suspensions. After 24 h, the zone of inhibition (ZOI) was estimated by the distance between the outer diameter of the inhibition and the diameter of the hydrogel. Besides, the obtained bacterial suspensions were cocultured with NH hydrogels. After 24 h, bacterial suspension samples were removed and stained by SYTO and propidium iodide (PI) for live/dead staining.

#### 2.2.4. Biocompatibility of the NH Hydrogels

Mouse fibroblasts (L929 cells) were used to coculture with NH hydrogels to evaluate the biocompatibility of the hydrogels. Briefly, L929 cells were first mixed in RPMI 1640 medium for recovery. When the cells proliferated stably, they were passaged and subsequently transferred to 24-well plates at a concentration of 105/mL to continue the culture. All cultured cells were randomly divided into control group (well plates containing culture medium) and different experimental groups (well plates containing culture medium and different NH hydrogels). The cell viability was observed by fluorescent staining when cocultured for 48 hours.

#### 2.2.5. In Vivo Therapeutic Effects of the NH Hydrogels

The therapeutic effect of NH hydrogels on infected wounds in the practical application was tested by establishing an infected wound model in SD rats. In brief, rats were first anesthetized by intraperitoneal injection of sodium pentobarbital (40 mg/kg body weight). The dorsal skin was then removed, and a mixed bacterial suspension containing Escherichia coli and Staphylococcus aureus (1 : 1) was added dropwise to the skin defect to form infected wounds. The modelled rats were randomly divided into three groups and treated with PBS, NH (0) hydrogel, and NH (500) hydrogel, respectively. Wound healing was recorded during the experiment. One week later, after the rats were anesthetized, the wound tissue was removed and immersed in 4% paraformaldehyde solution for subsequent pathological examination. Finally, the rats were sacrificed.

The obtained tissue samples were first sealed into wax blocks. The wax blocks were then processed into a series of sections for HE staining, Masson's trichrome staining, and immunohistochemical staining for inflammatory factors, including Interleukin 6 (IL-6) and tumor necrosis factor-*α* (TNF-*α*) and the vascular endothelial cell marker platelet endothelial cell adhesion molecule-1 (CD31).

#### 2.2.6. Statistical Analysis

Data from the various control and experimental groups were all assessed using Student's *t*-test and expressed as mean ± SD. ^*∗*^*p*  <  0.05 and *#p*  <  0.05 were considered statistically significant.

## 3. Results and Discussion

### 3.1. Preparation and Characterization of the NH Hydrogels

**Figure 1:**
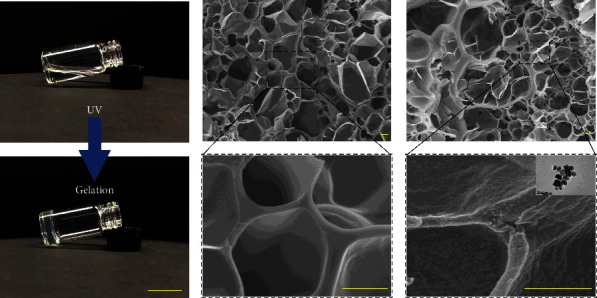
(a) Photos of the NH hydrogel formation process under UV. (b) SEM micrographs of NH hydrogel without Ag NPs. (c) SEM images of NH hydrogel with Ag NPs, inset shows the transmission electron microscopy (TEM) images of Ag NPs. The scale bar is 2 cm in (a), 10 *μ*m in (b), and 500 nm in (c).

In a typical experiment, the NH hydrogel system was firstly created by mixing N-acryloyl glycinamide monomer and Ag NPs solution at room temperature to form a pregel solution. Then, photoinitiator Irgacure 2959® was added to the monomer solution and vortexed vigorously. Subsequently, the binary supramolecular polymer hydrogels comprising NAGA were prepared via photoinitiated polymerization (Figure S1, Supplementary Materials). When subjected to UV stimulation, the mixture turns into a transparent hydrogel due to the formation of multiple hydrogen bonding domains between the dual amide in the N-acryloyl glycinamide side chain (Figure 1(a)). From the microstructure, the NH hydrogels had a rich network structure inside (Figure 1(b)), which facilitates their applications in areas such as active substance carriage. As shown in Figure 1(c), the sparse pore structure enabled the effective piggybacking of nanomaterials, Ag NPs. The Ag NPs have been widely used in medical, food, electronic, and other fields because of their high safety, good durability, and no drug resistance. The encapsulation of Ag NPs would make the hydrogel excellent antibacterial properties.

### 3.2. Mechanical and Self-Healing Performance of the NH Hydrogels

**Figure 2:**
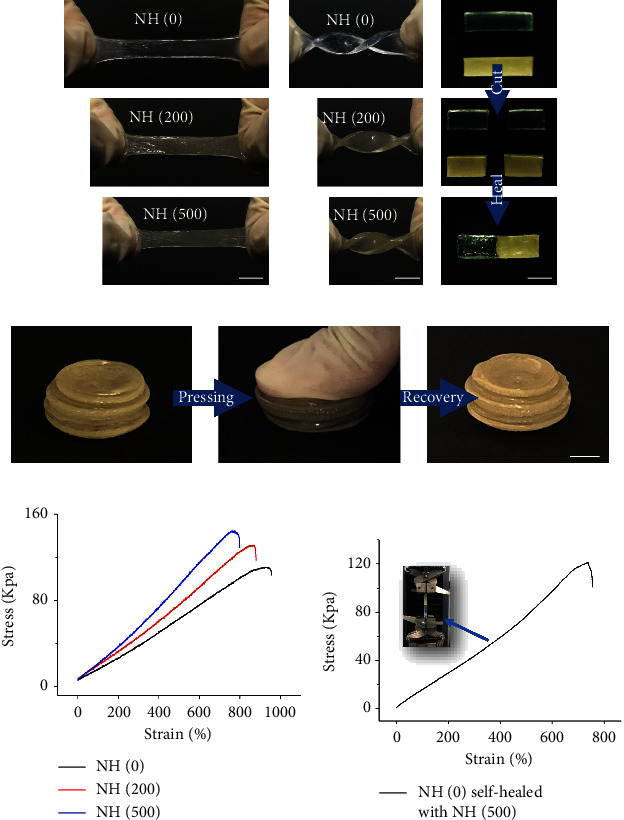
Deformations and mechanical performances of NH hydrogels: (a) stretching, (b) twisting and stretching, (c) self-healing, and (d) pressing and recovery. Tensile stress–strain curves of NH hydrogels: (e) pristine NH hydrogels and (f) self-healed NH hydrogels. The scale bars are 1 cm in (a), (b), and (c). The scale bar is 2 cm in (d).

Considering the inevitable damage during use can lead to rupture or even fracture of the hydrogel, which eventually leads to the failure of the hydrogel. Therefore, the prepared NH hydrogels were subjected to a series of mechanical tests. The hydrogel is strong enough to withstand knotting and stretching without any damage. And, it was found that the stretching properties of the hydrogel changed with the introduced content of Ag NPs (Figures 2(a) and 2(b)). Moreover, hydrogen bonding has also been used to produce self-healing hydrogels. As shown in Figure 2(c), two split separate hydrogel parts can be completely healed to form a new hydrogel in 2 min. Next, we assembled the same circular hydrogels together to form a cylindrical hydrogel. It was found that the healed cylindrical hydrogel is strong enough to withstand compression without any broken (Figure 2(d)). Furthermore, we examined the tensile strength of the NH hydrogels to analyse their mechanical properties and self-healing effects. It was found that the tensile strength of the NH hydrogels increased with the growth of Ag NPs content in the hydrogel system, but the stretchability weakened (Figure 2(e)). It was shown that the Ag NPs as fillers could increase the strength but also sacrifices the stretchability of the hydrogel, as a result. In addition, it was found that the healed hydrogel also had excellent tensile strength and deformability (Figure 2(f)). The above results confirmed the excellent mechanical and self-healing properties of NH hydrogels.

### 3.3. Antibacterial Activity of the NH Hydrogels

**Figure 3:**
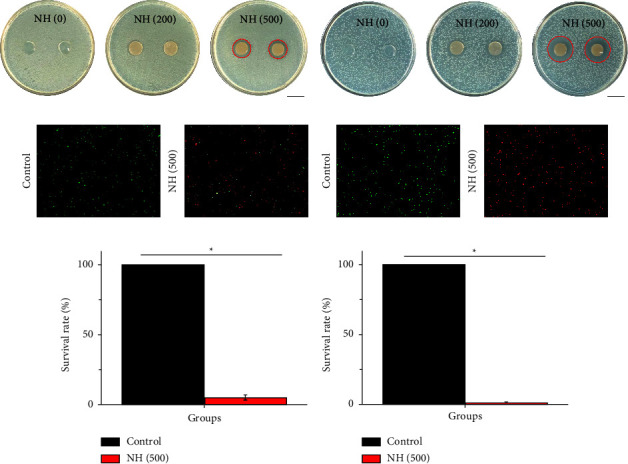
Optical images of the antibacterial activity of different NH hydrogels against (a) *E. coli* and (b) *S. aureus*. Live/dead staining of (c) *E. coli* and (d) *S. aureus*. The survival rate of (e) *S. aureus* and (f) *E. coli*. The scale bars in *a* and *b* are 200 *μ*m. The scale bar in *c* is 10 *μ*m.

Due to the antibacterial advantage of Ag NPs, the antibacterial activity of NH hydrogels was first investigated through the inhibition zone (Figures 3(a) and 3(b)). The results showed that there was absence of the zone of inhibition (ZOI) in the NH (0) group. The ZOI started to appear in the NH (200) group and pronounced in the NH (500) group (the ZOI was marked by a red circle). The best ZOI was in NH (500) hydrogels. The NH (500) hydrogel against *S. aureus* and *E. coli* was 27.21 ± 0.45 mm and 20.41 ± 0.27, respectively (Table S1, Supplementary Materials). These results indicated that the antibacterial efficiency of NH hydrogels increased with the amount of Ag NPs introduced. Furthermore, the bactericidal effect of NH (500) hydrogel was testified by the live-dead staining method. The fluorescence images and statistical results of live and dead bacteria showed that the NH (500) hydrogel showed great clearance of *S. aureus* and *E. coli* (Figures 3(c)–3(f)). It was found that the kill rate of NH (500) hydrogel against *S. aureus* and *E. coli* was 99.2 ± 0.62 and 96.42 ± 2.44, respectively (Table S2, Supplementary Materials). This feature shows the promise of the NH hydrogels for the treatment of infected wounds.

### 3.4. Biocompatibility of the NH Hydrogels

**Figure 4:**
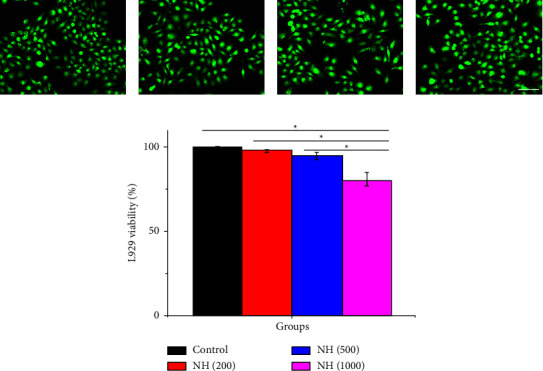
Fluorescence images of L929 cells under different culture conditions, including (a) pure culture medium, (b) culture medium with NH (200) hydrogel, (c) culture medium with NH (500) hydrogel, and (d) culture medium with NH (1000) hydrogel. (e) The L929 viability cultured in medium with or without different NH hydrogels. The scale bar is 40 *μ*m.

With the increased content of Ag NPs, the NH hydrogel system was endowed with an antibacterial capacity with its gain but also with an increased risk of biosafety. Therefore, it is important to study their biocompatibility before using them as wound dressings. We selected L929 cells to cocultured with NH hydrogels and assessed their biocompatibility by observing the activity of the cells during the culture, as shown in Figure 4. It was found that the cells cocultured in NH (200) and NH (500) hydrogel groups had similar morphology and proliferation as the normal control group while the NH (1000) hydrogel coculture system showed cell death (Figures 4(a)–4(d)). The same results were obtained from the cell viability statistics (Figure 4(e)). This indicates that the introduction of excessive Ag NPs into NH hydrogels could cause significant side effects, which are rather detrimental to the in vivo application of NH hydrogels.

### 3.5. The In Vivo Effect of the NH Hydrogels

**Figure 5:**
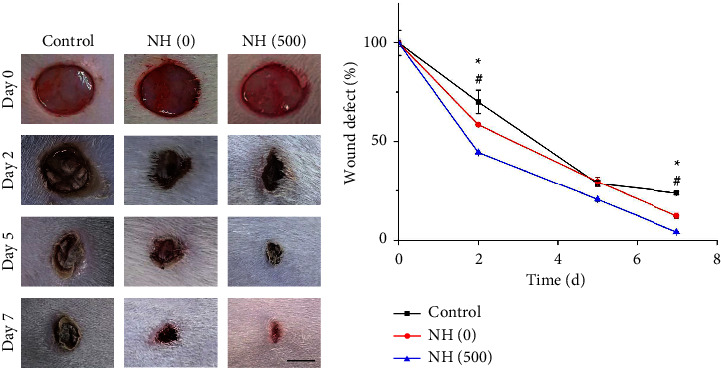
(a) The wound images of the control group, NH (0) group, and NH (500) group on day 0, 2, 5, 7, respectively. (b) The relative wound healing curve of (a). The scale bar is 5 mm in (a).

Since NH (500) hydrogel exhibited great mechanical, antibacterial, and biocompatible properties, we further observed its effect on infected wound healing. To demonstrate this, we modelled infected wounds in rats and treated the wounds with PBS solution, NH (0) hydrogel, and NH (500) hydrogel, respectively. After an operation, the recovery of the wounds was recorded at specific time points during the week, as shown in Figure 5(a). The results showed that on the second day after injury, the wound tissue in the control group developed significant swelling with exudate. Although infection also occurred in the NH (0) and NH (500) groups, it appeared to be better than in the control group. At the end of the experiment, the NH (500) group showed the best efficacy with the best wound healing (Figure 5(b)). The reason for this may be due to the synergistic effect of the hydrogel dressing as a barrier combined with the antibacterial properties of the Ag NPs. It was followed by the NH (0) group, and the worst was the control group. Because despite the lack of infection control in the NH (0) group, it still exhibited a beneficial effect, creating an appreciable wound recovery.

**Figure 6:**
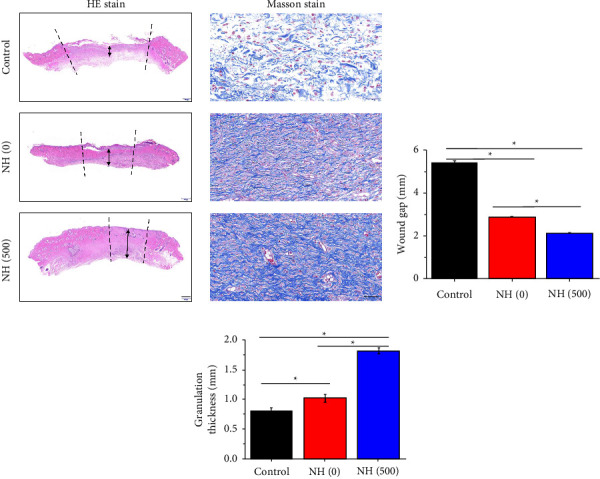
(a) HE and Masson staining of the wound tissues from control, NH (0), and NH (500) group. (b), (c) The wound gap and granulation thickness of the wound tissues from control, NH (0), and NH (500) groups. The wound gap was marked by two black dashed lines, and the thickness of the granulation was marked by a black arrow in (a). The scale bar is 500 *μ*m in HE stains and 50 *μ*m in Masson stains in (a).

To further analyse the recovery of the wound, we first analysed the granulation tissue and collagen deposition at the wound site. From the results of HE staining, among all groups, it was found that the granulation tissue in the NH (500) hydrogel group had a complete and regular structure, which was the closest to the structure of normal skin tissue (Figure 6(a)). In addition, from the results of collagen deposition, the NH (500) hydrogel group had the highest collagen fibre content and orderly collagen fibre arrangement, indicating that the wound tissue recovered well and was in the remodelling stage (Figure 6(a)). Further, the statistical results of the wound gap and the thickness of the granulation tissue showed that the control group healed the worst with the largest wound defect and the thinnest thickness of the granulation tissue, the NH (0) hydrogel group slightly better, and the NH (500) hydrogel group the best (Figures 6(b) and 6(c)).

**Figure 7:**
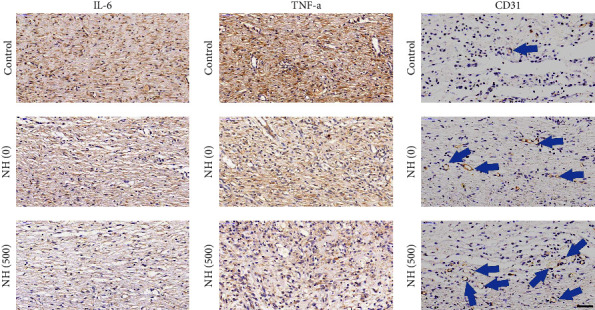
Immunohistochemical detection of inflammatory factors and angiogenesis. (a) IL-6, (b) TNF-*α*, and (c) CD31, and the vascular structures were indicated by blue arrows. The scale bar is 50 *μ*m.

Excessive activation of the inflammatory response due to infection can hinder the wound repair process. Therefore, we tested the proinflammatory factors IL-6 and TNF-*α* to assess the infection of the wound (Figures 7(a) and 7(b)). Among groups, it was found that the control group had the highest positive expression of IL-6 and TNF-*α*, indicating the presence of a persistent infection resulting in high levels of inflammation. In contrast, NH (500) hydrogel-treated wounds had low amounts of IL-6 and TNF-*α*, indicating that the infection was well controlled. Notably, neovascularization is highly relevant for tissue development and maturation and is a key indicator for the analysis of wound healing. Therefore, we analysed the distribution of the vascular endothelial cell marker CD31 in the tissue samples from different groups (Figure 7(c)). As well, it was found the highest distribution of neovascularization in the NH (500) hydrogel group. The NH (0) hydrogel group, which had only a limited protective effect due to lack of ability to deal with infection, had a slightly better number of angiogenesis than the control group, which lacked any protective measures. These results indicated that the NH hydrogels have a great prospect of application in the direction of infected wound treatment.

## 4. Conclusion

In summary, we developed an antimicrobial self-healing supramolecular hydrogel for the treatment of infected wounds. The NH hydrogel is simple and convenient to prepare because a pregel solution mixed with N-acryloylglycine and Ag NPs could be polymerized under light. The NH hydrogel network forms extensive hydrogen bonds thanks to the diamide group of the NAGA monomer and thus exhibits significant mechanical demonstration and self-healing properties. On the other side, the NH hydrogel inherited good antimicrobial properties due to the introduction of Ag NPs. In addition, the hydrogel had good biocompatibility, which allows it to be used in several in vivo studies. In this study, the NH hydrogel had been adopted as a dressing for the treatment of infected wounds. The NH hydrogel had been shown to promote inflammation relief, collagen deposition, and neovascularization. Therefore, the NH hydrogels have a great prospect of application in the direction of infected wound treatment.

## Data Availability

The raw/processed data required to reproduce these findings cannot be shared at this time as the data also form part of an ongoing study.

## Supplementary Materials

Supplementary Materials Figure S1: the schematic images of the polymerization of the binary supramolecular polymer hydrogels. Table S1: the ZOI results of the NH hydrogels against different strains. Table S2: the results of the NH (500) hydrogels against different strains.
