# Supplementary material for: Self-Healing Supramolecular Hydrogels with Antibacterial Abilities for Wound Healing
Source: J Healthc Eng. 2023 Feb 9;2023:7109766. doi: 10.1155/2023/7109766 (PMC9935882; doi:10.1155/2023/7109766)
Supplement: Supplementary Materials — Figure S1: the schematic images of the polymerization of the binary supramolecular polymer hydrogels. Table S1: the ZOI results of the NH hydrogels against different strains. Table S2: the results of the NH (500) hydrogels against different strains. [file 7109766.f1.docx]

Supplementary Materials

Self-Healing Supramolecular Hydrogels with Anti-Bacterial Abilities for Wound Healing

Zhiwu Hong^1^, Lei Wu^1^, Zherui Zhang^1^, Jinpeng Zhang^1^, Huajian Ren^1^, Gefei Wang^1^, Xiuwen Wu^1^, Guosheng Gu^1,2,*^, Jianan Ren^1,*^

*^1^Research Institute of General Surgery, Jinling Hospital, Nanjing Medical University, Nanjing, China*

*^2^Department of General Surgery, Anhui No.2 Provincial Peoples’ Hospital, Anhui, China*

*Corresponding author: Guosheng Gu, Email: guguoshengde@163.com, Address: Department of Surgery, Jinling Hospital, 305 East Zhongshan Road, Nanjing, 210002, China.

Jianan Ren, Email: jiananr@gmail.com, Address: Department of Surgery, Jinling Hospital, 305 East Zhongshan Road, Nanjing, 210002, China.


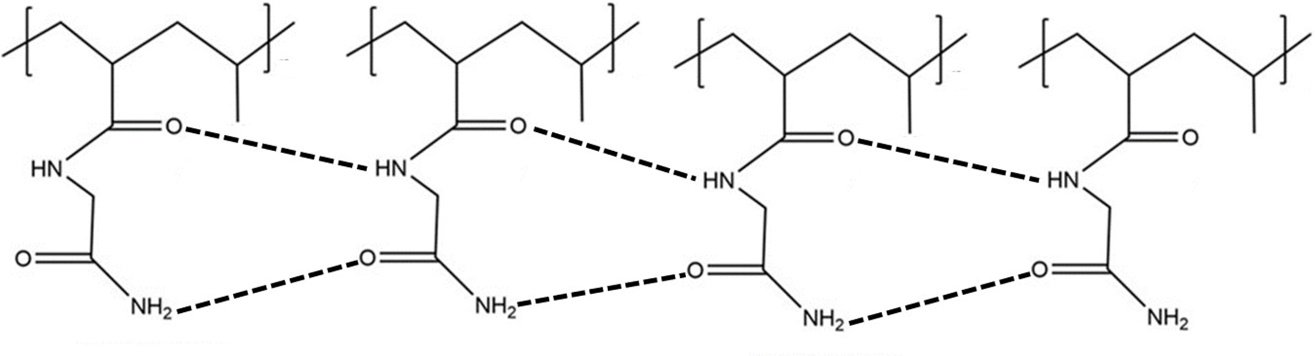


Figure S1. The schematic images of the polymeration of the binary supramolecular polymer hydrogels.

**Talble S1** The ZOI results of the NH hydrogels against different strains.

| Strains | NH (0) | NH (200) | NH (500) |
| --- | --- | --- | --- |
| Escherichia coli | 0 | 16.73±0.13 | 20.41±0.27 |
| Staphylococcus aureus | 0 | 19.49±0.17 | 27.21±0.45 |

**Talble S2** The results of NH (500) hydrogels against different strains.

| Strains | Kill (%) |
| --- | --- |
| Escherichia coli | 96.42±2.44 |
| Staphylococcus aureus | 99.2±0.62 |
